# Nintedanib preserves lung growth and prevents pulmonary hypertension in a hyperoxia-induced lung injury model

**DOI:** 10.1038/s41390-024-03562-0

**Published:** 2024-10-11

**Authors:** Kathy L. Ding, Caroline Smith, Gregory Seedorf, Steven H. Abman

**Affiliations:** 1 Medical Student Research Track, University of Colorado School of Medicine, Aurora, CO USA; 2 Pediatric Heart Lung Center, Department of Pediatrics, University of Colorado School of Medicine, Aurora, CO USA

## Abstract

**Background:**

Bronchopulmonary dysplasia (BPD), the chronic lung disease associated with prematurity, is characterized by poor alveolar and vascular growth, interstitial fibrosis, and pulmonary hypertension (PH). Although multifactorial in origin, the pathophysiology of BPD is partly attributed to hyperoxia-induced postnatal injury, resulting in lung fibrosis. Recent work has shown that anti-fibrotic agents, including Nintedanib (NTD), can preserve lung function in adults with idiopathic pulmonary fibrosis. However, NTD is a non-specific tyrosine kinase receptor inhibitor that can potentially have adverse effects on the developing lung, and whether NTD treatment can prevent or worsen risk for BPD and PH is unknown.

**Hypothesis:**

We hypothesize that NTD treatment will preserve lung growth and function and prevent PH in an experimental model of hyperoxia-induced BPD in rats.

**Methods:**

Newborn rats were exposed to either hyperoxia (90%) or room air (RA) conditions and received daily treatment of NTD or saline (control) by intraperitoneal (IP) injections (1 mg/kg) for 14 days, beginning on postnatal day 1. At day 14, lung mechanics were measured prior to harvesting lung and cardiac tissue. Lung mechanics, including total respiratory resistance and compliance, were measured using a flexiVent system. Lung tissue was evaluated for radial alveolar counts (RAC), mean linear intercept (MLI), pulmonary vessel density (PVD), and pulmonary vessel wall thickness (PVWT). Right ventricular hypertrophy (RVH) was quantified with cardiac weights using Fulton’s index (ratio of right ventricle to the left ventricle plus septum).

**Results:**

When compared with RA controls, hyperoxia exposure reduced RAC by 64% (*p* < 0.01) and PVD by 65% (*p* < 0.01) and increased MLI by 108% (*p* < 0.01) and RVH by 118% (*p* < 0.01). Hyperoxia increased total respiratory resistance by 94% and reduced lung compliance by 75% (*p* < 0.01 for each). NTD administration restored RAC, MLI, RVH, PVWT and total respiratory resistance to control values and improved PVD and total lung compliance in the hyperoxia-exposed rats. NTD treatment of control animals did not have adverse effects on lung structure or function at 1 mg/kg. When administered at higher doses of 50 mg/kg, NTD significantly reduced alveolar growth in RA controls, suggesting dose-related effects on normal lung structure.

**Conclusions:**

We found that NTD treatment preserved lung alveolar and vascular growth, improved lung function, and reduced RVH in experimental BPD in infant rats without apparent adverse effects in control animals. We speculate that although potentially harmful at high doses, NTD may provide a novel therapeutic strategy for prevention of BPD and PH.

**Impact:**

- Anti-fibrotic therapies may be a novel therapeutic strategy for the treatment or prevention of BPD.
- High-dose anti-fibrotics may have adverse effects on developing lungs, while low-dose anti-fibrotics may treat or prevent BPD.
- There is very little preclinical and clinical data on the use of anti-fibrotics in the developing lung.
- Dose timing and duration of anti-fibrotic therapies may be critical for the treatment of neonatal lung disease.
- Currently, strategies for the prevention and treatment of BPD are lacking, especially in the context of lung fibrosis, so this research has major clinical applicability.

## Introduction

Despite marked advancements in therapeutics directed towards the survival of preterm neonates in past decades, the incidence of bronchopulmonary dysplasia (BPD), the chronic lung disease of prematurity, continues to be one of the most frequent causes of morbidity and mortality in preterm infants.^1,2^ The prevalence of BPD has been steadily increasing due to rising survival of extremely premature infants with the use of antenatal corticosteroids, more effective respiratory support, and utilization of pulmonary surfactant. Despite improvement in many co-morbidities associated with prematurity the incidence of BPD in premature neonates born before 29 weeks gestational age remains at approximately 40%^3–5^ and preventive strategies are lacking.^6,7^

The pathophysiology of BPD is multifactorial in origin and stems from a complex interplay between antenatal and postnatal risk factors.^8,9^ One critical postnatal factor that contributes to the development of BPD is exposure of the fragile preterm lung to hyperoxia, which results in lung injury due to the formation of reactive oxygen species during administration of supplemental oxygen to manage hypoxic respiratory failure of severely preterm infants.^6,7,10^ Although clearly multifactorial in etiology, BPD and late cardiorespiratory sequelae including pulmonary hypertension (PH), reflects the interplay between prematurity, exposure to injurious stimuli as well with aberrant or insufficient lung repair mechanisms in response to early injury that ultimately causes arrest of alveolarization and angiogenesis, with pathologic remodeling of the pulmonary microvasculature.^6^ Poor repair processes following this pro-inflammatory response within the lung eventually results in fibrosis of alveolar mesenchyme, scarring of lung tissue, and abnormal lung mechanics that persist into adulthood.^6,11^ About 25% of neonates with moderate-severe BPD develop pulmonary hypertension (PH) as well, which is associated with increased mortality due to development of right heart failure from elevated pulmonary vascular resistance.^12^ The long-term complications prove to be detrimental, but treatments for the reversal of BPD-associated fibrosis and PH continue to be sparse.^12,13^

Recent studies have shown that anti-fibrotic agents, including Nintedanib, can preserve lung function in adults with interstitial lung diseases, particularly idiopathic pulmonary fibrosis (IPF).^14–16^ IPF is also characterized by fibrosis and excessive extracellular matrix and collagen deposition via unregulated proliferation of myofibroblasts.^15–17^ Like BPD, the pathogenesis of IPF involves defective lung repair processes in response to repetitive alveolar epithelium injury. Nintedanib, one of the first FDA-approved anti-fibrotics for the treatment of IPF in adults, acts through inhibition of tyrosine kinase receptors, which results in blockade of diverse signaling cascades that regulate cell proliferation and migration.

Experimental studies have demonstrated the efficacy of Nintedanib in attenuating myofibroblast transformation and significantly reducing IPF disease progression.^15,17–19^ Given the similarities between the pathogenesis of BPD and IPF, we considered the applicability of using Nintedanib towards the treatment of BPD; however, because Nintedanib exerts its effect on a wide range of growth factor receptor signals,^18,19^ it has the potential to adversely affect lung growth by disrupting multiple pathways involved with normal lung development, including vascular endothelial growth factor (VEGF), platelet-derived growth factor (PDGF), fibroblast growth factor (FGF) and others.^17–19^ Past work has shown that even brief inhibition of VEGF or PDGF in the perinatal period inhibits alveolarization and impairs subsequent lung development throughout infancy.^20,21^ Whether Nintedanib treatment has adverse effects on normal lung development in neonates or can prevent BPD by preserving lung alveolar and vascular growth and preventing PH is unknown.

Therefore, we propose the hypothesis that Nintedanib treatment will preserve lung alveolar and vascular growth, improve lung function, and prevent PH in a hyperoxia-induced BPD model in infant rats.^22^ To test this hypothesis, we studied dose-related effects of Nintedanib treatment on lung growth in normal neonatal rats. We further studied the effects of Nintedanib treatment on lung structure and function and the severity of PH in infant rats during exposure to hyperoxia.

## Methods

### Animal protocols

All protocols and procedures were approved by the Animal Care and Use Committee at the University of Colorado Anschutz Medical Center.

### Study design

Dose-ranging studies for Nintedanib were initially conducted with healthy, control Sprague Dawley rats to establish an appropriate starting dose. On day of life 1, the rats were randomly assigned to receive daily intraperitoneal injections of Nintedanib doses at 1 mg/kg, 10 mg/kg, or 50 mg/kg. The 50 mg/kg and 10 mg/kg Nintedanib doses were found to be significantly lethal, and all rats were sacrificed by day 5 for tissue harvest. Rats receiving Nintedanib treatment at the 1 mg/kg dose survived to day 14 before being sacrificed for tissue collection. No adverse lung effects were seen in healthy rats receiving the 1 mg/kg Nintedanib injections, so this dose was selected to be the treatment dose for the hyperoxia studies.

For the hyperoxia studies, Sprague Dawley rats were randomly assigned on day of life 1 to be exposed to either room air (RA) or hyperoxia conditions for 14 days. In the postnatal hyperoxia model, pups were placed into hyperoxia chambers within 30 minutes after birth. The oxygen concentration in the chambers was maintained at Fi_O2_ = 0.90 (Po_2_ = 466 mmHg at Denver’s altitude with barometric pressure = 630 mmHg, which is equivalent to an Fi_O2_ of 0.74 at sea level) for 14 days.^23^ During this period, the rats in both groups received either daily subcutaneous injection of normal saline for the control group or Nintedanib at 1 mg/kg for the treatment group.

### Lung mechanics

At day 14, lung mechanics were measured with a flexiVent system (Flexivent; SCIREQ, Montreal, QC, Canada) in anesthetized rats to measure total lung resistance and compliance according to standard methods. Fourteen-day-old rats were anesthetized with ketamine, and tracheostomy and cannulation were performed. Rats were subsequently placed on mechanical ventilation and lung metrics were determined.

### Tissue collection

Following measurement of lung mechanics, rats were sacrificed with intraperitoneal pentobarbital sodium. Lungs were immersed in 4% paraformaldehyde at room temperature and maintained at 20 cm H2O pressure for 60 minutes. Lungs were immersed in 4% paraformaldehyde for overnight fixation, and two sections from each animal were paraffin embedded for histologic analysis. Cardiac tissue (right ventricle, left ventricle, and septum) was also collected for cardiac weight assessment as an indicator of pulmonary hypertension.

### Immunohistochemistry

Slides with 5-mm paraffin sections were stained with hematoxylin and eosin for assessment of alveolar structure by morphometric analysis and von Willebrand Factor (vWF), an endothelial cell-specific marker, to assess pulmonary vessel density.

### Morphometric analysis

Mean linear intercepts (MLI), radial alveolar counts (RAC), pulmonary vessel density (PVD), pulmonary vascular wall thickness (PVWT), and right ventricular hypertrophy (RVH) were determined by standard morphometric techniques.^23–27^ MLI and RAC were used as indices for alveolar structural integrity. Pulmonary vessel density was calculated by counting vWF-stained vessels with 50 µm or less per high-powered field. Pulmonary vessel wall thickness was also determined on those with external diameter with 50 µm or less per high-powered field using computer-assisted image analysis software. In each animal, at least five measurements were obtained for MLI, RAC, PVD, and PVWT, at least 10 images were processed for computer-assisted analysis of alveolar structure, and at least 10 pulmonary vessels were measured for pulmonary vessel density and vascular wall thickness. RVH was quantified by measuring cardiac weights of the right ventricle (RV) and left ventricle plus septum (LV + S), and Fulton’s index was calculated (RV/(LV + S).

### Western blot analysis

Lung tissue for western blot analysis was harvested on day 5 to identify whether Nintedanib would exert mechanistic changes on signaling pathways in early lung development. The lungs were subsequently frozen for protein collection. The frozen lung samples were subject to western blot analysis of phosphorylated Src kinase as well as total Src kinase per standard methods, imaged by chemilumiescence with Amersham Imagequant 800 and analyzed with ImageLab software. For the blots, β-actin was utilized as a housekeeping protein to compare expression levels of between samples. Densitometry was used to quantify protein levels.

### PCR analysis

Rats utilized for PCR analysis were also sacrificed on day-of-life 5 to identify whether Nintedanib would influence inflammatory and apoptotic markers earlier on in lung development. Inflammatory cytokines, including IL-1a and TNFa, and the apoptotic marker Caspase 3 were assessed for gene expression using TaqMan RT-qPCR analysis per standard protocol on Applied Biosystems ViiA7.

### Statistical analysis

Statistical analysis was performed with the GraphPad Prism 8.0 software package (GraphPad Software, San Diego, CA). Statistical comparisons were made between groups using t-test or analysis of variance with Kruskal-Wallis/Dunns post-hoc analysis for significance. A *p*-value < 0.05 was considered significant. Data are presented as mean +/- standard error.

## Results

**Fig. 1:**
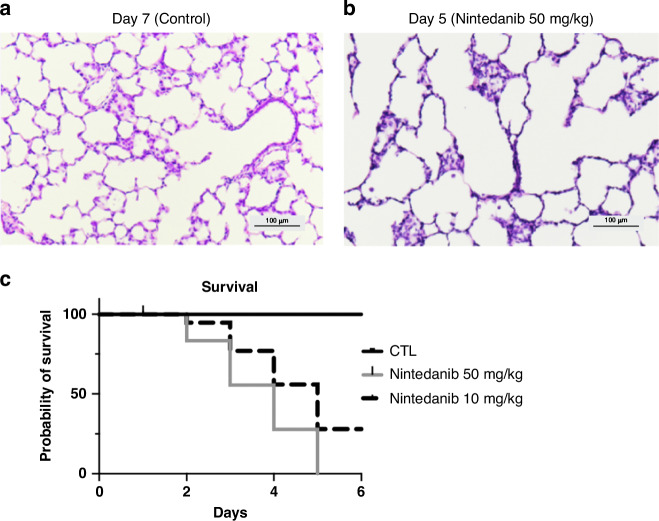
Histology and survival curves for control vs Nintedanib treated rats. Histological comparisons of preserved, alveolar structure in healthy, control rats on day of life 7 (**a**) with severe alveolar simplification seen in healthy rats treated with Nintedanib at a dose of 50 mg/kg on day of life 5 (**b**) are shown here. The survival graph (**c**) demonstrates the survival curves of healthy, control rats compared with healthy rats treated with Nintedanib at a dose of 10 mg/kg and at a dose of 50 mg/kg. Rats treated with 10 mg/kg of Nintedanib experienced 72% mortality by day of life 5. Rats treated with 50 mg/kg of Nintedanib experienced 100% mortality by day of life 5. Control rats experienced no mortality.

For the dose-ranging studies conducted under normoxia conditions, survival and alveolar structure were studied in control rat pups who received Nintedanib treatment at 1 mg/kg, 10 mg/kg, and 50 mg/kg doses. Control rats demonstrated preserved alveolar structure and had no deaths (Fig. 1a, c), while rats treated with daily Nintedanib administration at both 50 mg/kg and 10 mg/kg had severely impaired lung growth, as evidenced by the dilated distal airspaces with reduced septations (Fig. 1b). The survival curves (Fig. 1c) further highlight that rats treated with 10 mg/kg of Nintedanib experienced 72% mortality by day of life 5 while rats treated with 50 mg/kg of Nintedanib experienced 100% mortality on day of life 5. Lung structure was normal in rats exposed to the lower Nintedanib dose of 1 mg/kg dose and there were no deaths.

**Fig. 2:**
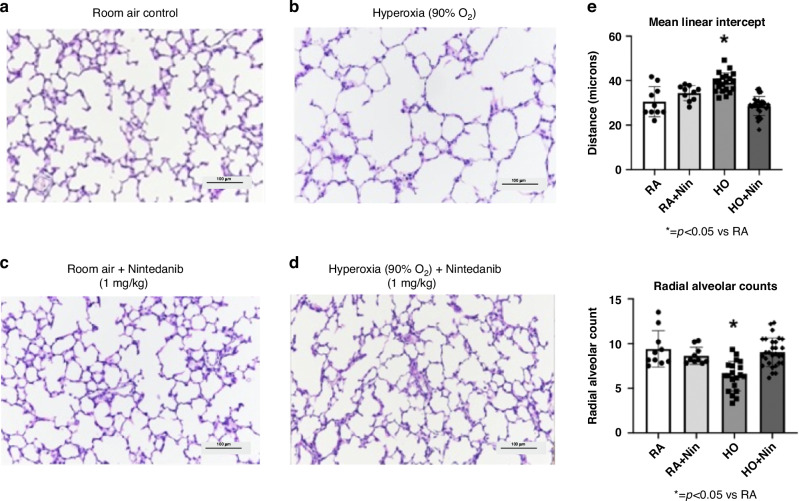
Nintedanib treatment at 1 mg/kg restored alveolar structure in hyperoxia-exposed rats. Histological representations of lung structure in saline control rats (**a**), postnatal hyperoxia-exposed rats (**b**), control rats treated with 1 mg/kg of Nintedanib (**c**), and hyperoxia-exposed rats treated with 1 mg/kg of Nintedanib (**d**) are shown here. Nintedanib treatment was found to prevent alveolar simplification in rats subject to hyperoxia exposure as compared to controls and had no visible adverse effects on alveolarization in healthy, control rats. The micrographs (**e**) demonstrate that compared with the control rats, postnatal hyperoxia exposure reduced RAC by 64% (*p* < 0.01) and increased MLI by 108% (*p* < 0.01). Nintedanib treatment at 1 mg/kg of hyperoxia-exposed rats was found to significantly restore both RAC and MLI back to control values. (RA Control: *n* = 10, RA+Nintedanib: *n* = 10, hyperoxia-exposed: *n* = 20, hyperoxia-exposed+Nintedanib: *n* = 29).

To determine the effects of Nintedanib treatment in experimental BPD, we compared the effects of Nintedanib (1 mg/kg/day) treatment with daily saline injections (controls) on lung structure and function and PH as assessed by RVH in infant rats exposed to either room air (RA) or postnatal hyperoxia (90% O_2_) conditions. After 2 weeks, saline-treated control rats in RA had histologically normal lung structure (Fig. 2a). In comparison, rat pups that were exposed to postnatal hyperoxia conditions demonstrated histological evidence of marked alveolar simplification with dilated distal airspaces and reduced septations (Fig. 2b). Rats exposed to room air and treated with Nintedanib (1 mg/kg/day) for 14 days had normal alveolar structure (Fig. 2c). Of note, none of the rats in the hyperoxia group not treated with Nintedanib experienced mortality before 14 days. The data was also analyzed for sex-related differences for all the following measurements and no differences were identified.

**Fig. 3:**
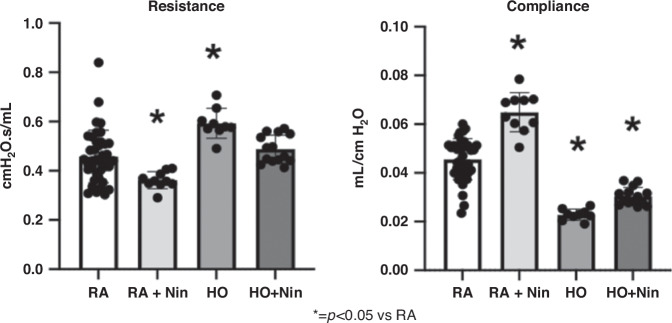
Micrographs demonstrate that compared to RA controls, rats from the hyperoxia group had increased total respiratory system resistance by 94% (*p* < 0.01) and reduced total respiratory system compliance by 75% (*p* < 0.01). Nintedanib treatment at 1 mg/kg of hyperoxia-exposed rats was found to significantly restore total respiratory resistance back to control values but did not significantly increase compliance. (RA control: *n* = 50, RA+Nintedanib: *n* = 20, hyperoxia-exposed: *n* = 19, hyperoxia-exposed+Nintedanib: *n* = 24).

Importantly, hyperoxia-exposed rats treated with Nintedanib (1 mg/kg) had significantly improved lung structure (Fig. 2d) in comparison with hyperoxia-exposed rats without treatment. When compared with the control rats, postnatal hyperoxia exposure reduced RAC by 64% (*p* < 0.01) and increased MLI by 108% (*p* < 0.01) (Fig. 2e). In rat pups exposed to postnatal hyperoxia, Nintedanib treatment preserved normal alveolar structure by histology and restored both RAC and MLI measurements back to control values (Fig. 2e). In comparison to control rats at day 14, rats from the hyperoxia group had increased total respiratory system resistance by 94% (*p* < 0.01) and reduced total respiratory system compliance by 75% (*p* < 0.01) (Fig. 3). Lung mechanics were improved in hyperoxia-exposed rats that were treated with Nintedanib, as reduced total respiratory system resistance was not different from control values (Fig. 3).

**Fig. 4:**
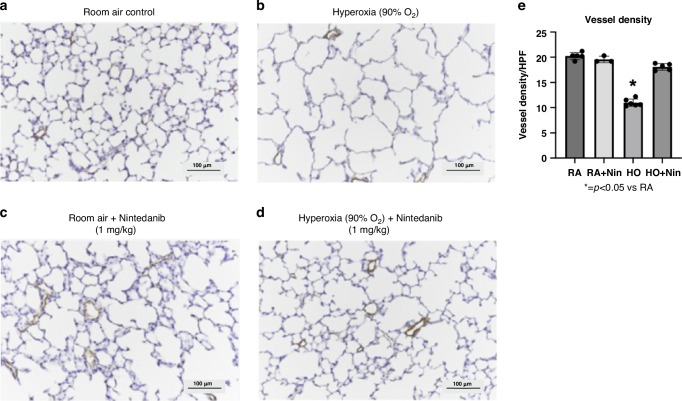
Nintedanib treatment at 1 mg/kg improved pulmonary vessel density in hyperoxia-exposed rats. Histological representations of pulmonary vasculature in in saline control rats (**a**), postnatal hyperoxia-exposed rats (**b**), control rats treated with 1 mg/kg of Nintedanib (**c**), and hyperoxia-exposed rats treated with 1 mg/kg of Nintedanib (**d**) are shown here. Nintedanib treatment visually preserved pulmonary vasculature in control rats and improved pulmonary vessel density in hyperoxia-exposed rats. The micrographs (**e**) demonstrate that compared to control rats, hyperoxia exposure significantly reduced PVD by 65% (*p* < 0.01). Nintedanib treatment at 1 mg/kg of hyperoxia-exposed rats was found to restore PVD back to control values. (RA control: *n* = 5, RA+Nintedanib: *n* = 3, hyperoxia-exposed: *n* = 6, hyperoxia-exposed+Nintedanib: *n* = 5).

In comparison with the pulmonary vessel density (PVD) histology of control rats (Fig. 4a), hyperoxia-exposed rats had markedly decreased PVD (Fig. 4b). Rats exposed to room air and treated with Nintedanib (1 mg/kg/day) for 14 days had PVD similar to controls (Fig. 4c), and hyperoxia-exposed rats treated with Nintedanib (1 mg/kg) had histologically increased PVD (Fig. 4d) in comparison with hyperoxia-exposed rats without treatment. There were no differences in PVD as measured in RA control rates treated with Nintedanib or saline. In comparison to controls, PVD was reduced by 65% in hyperoxia-exposed rats without treatment (*p* < 0.01). Nintedanib treatment of hyperoxia-exposed rats improved PVD to similar values as measured in controls (Fig. 4e).

**Fig. 5:**
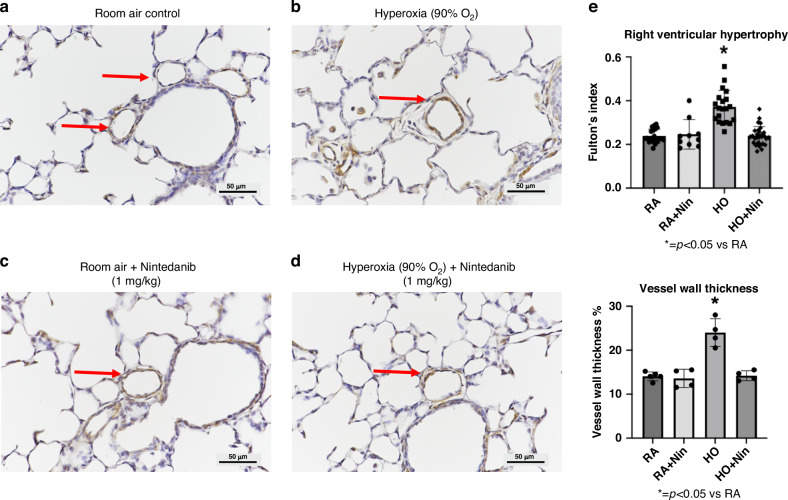
Nintedanib treatment at 1 mg/kg reduced pulmonary vessel wall thickness and RVH in hyperoxia-exposed rats. Histological representations of pulmonary vessel wall thickness in saline control rats (**a**), postnatal hyperoxia-exposed rats (**b**), control rats treated with 1 mg/kg of Nintedanib (**c**), and hyperoxia-exposed rats treated with 1 mg/kg of Nintedanib (**d**) are shown here. Vessels chosen for measurements are marked by red arrows. Nintedanib treatment visually preserved pulmonary vessel wall thickness in control rats and reduced the thickness in hyperoxia-exposed rats. The micrographs (**e**) demonstrate that hyperoxia significantly increased RVH by 118% (*p* < 0.01) and PVWT by 170% (*p* < 0.02) as compared to controls. Nintedanib treatment at 1 mg/kg of hyperoxia-exposed rats was found to significantly decrease pulmonary vessel wall thickness and RVH back to control values. (For RVH, RA control: *n* = 20, RA+Nintedanib: *n* = 10, hyperoxia-exposed: *n* = 20, hyperoxia-exposed+Nintedanib: *n* = 29). (For PVWT, RA control: *n* = 5, RA+Nintedanib: *n* = 4, hyperoxia-exposed: *n* = 4, hyperoxia-exposed+Nintedanib: *n* = 4).

Additionally, when comparing pulmonary vascular wall thickness (PVWT) histology of control rats (Fig. 5a) to PVWT histology of hyperoxia-exposed rats (Fig. 5b), the hyperoxia group had much thicker pulmonary vessel walls. Rats exposed to room air and treated with Nintedanib (1 mg/kg/day) for 14 days had PVWT similar to controls (Fig. 5c), and hyperoxia-exposed rats treated with Nintedanib (1 mg/kg) had a notable reduction in PVWT (Fig. 5d) compared with hyperoxia-exposed rats without treatment. Compared to controls, PVWT was elevated by 170% in hyperoxia-exposed rats without treatment (*p* < 0.02) and RVH was increased by 118% (*p* < 0.01). Quantitative analysis also found that Nintedanib treatment attenuated hyperoxia-induced increases in PVWT of small pulmonary arteries and prevented the development of RVH, restoring PVWT and RVH to control values (Fig. 5e).

**Fig. 6:**
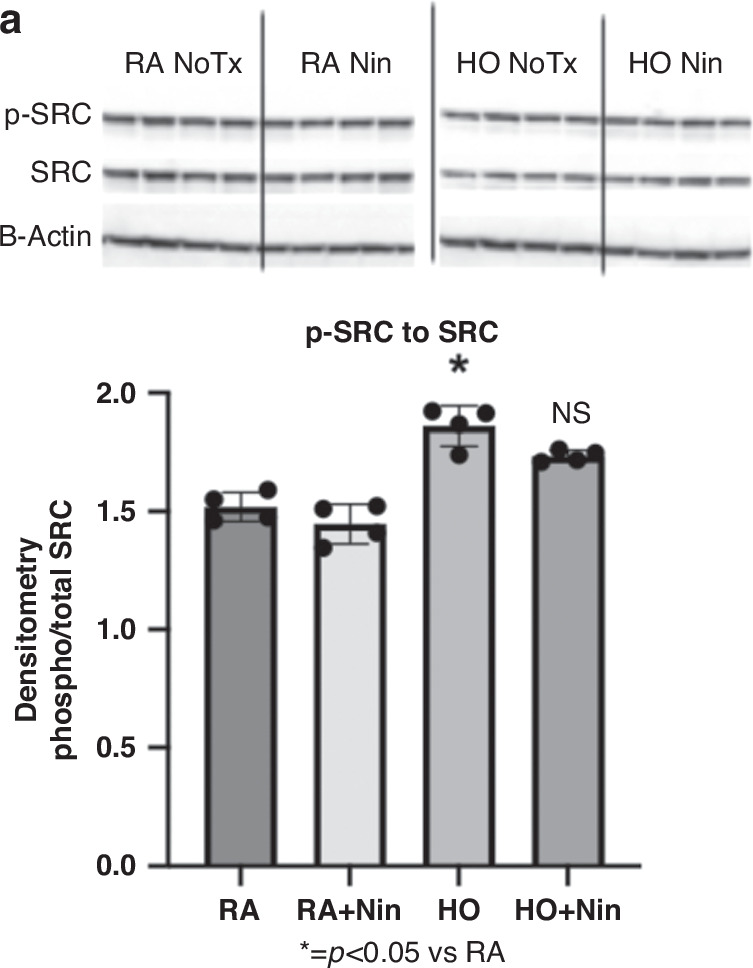
Protein expression detected by western blot analysis demonstrated that compared to RA controls, hyperoxia exposure increased phosphorylated Src by 122% (*p* < 0.02). Protein expression of day 5 lungs (**a**) showed no significant difference between phosphorylated Src kinase expression over total Src kinase expression in the hyperoxia-exposed group compared with the hyperoxia-exposed group that received Nintedanib treatment. (RA control: *n* = 4, RA+Nintedanib: *n* = 4, hyperoxia-exposed: *n* = 4, hyperoxia-exposed+Nintedanib: *n* = 4).

**Fig. 7:**
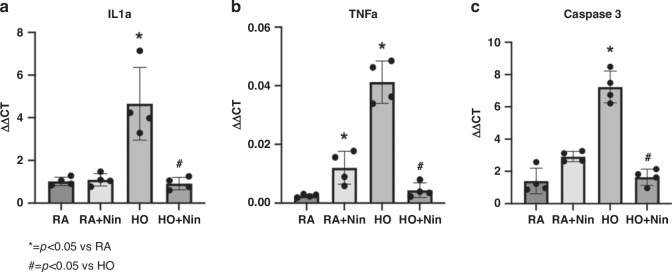
Inflammatory and apoptotic markers were reduced with Nintedanib treatment at 1 mg/kg. PCR analysis of gene expression in day 5 lungs showed  that inflammatory cytokines IL1a and TNFa were significantly elevated in the hyperoxia group compared with the RA control group by 452% (*p* < 0.05) and 668% (*p* < 0.01), respectively (**a**, **b**). The gene expression of apoptotic marker Caspase 3 (**c**) was also significantly elevated by 516% (*p* < 0.01) in the hyperoxia group compared with the RA control group (Fig. 7c). Nintedanib treatment of the hyperoxia group restored IL1a, TNFa, and Caspase 3 levels back to control values. (RA control: *n* = 4, RA+Nintedanib: *n* = 4, hyperoxia-exposed: *n* = 4, hyperoxia-exposed+Nintedanib: *n* = 4).

For the western blot analysis with densitometry comparing phosphorylated-Src kinase with total Src kinase (Fig. 6a), protein expression of phosphorylated-Src kinase was significantly elevated by 122% (*p* < 0.02) in the hyperoxia group in comparison to the RA control group. There was no significant change in protein expression of the phosphorylated-Src expression in the hyperoxia group treated with Nintedanib compared to the hyperoxia group without treatment. Regarding PCR analysis of inflammatory cytokines (IL1a, TNFa) and apoptotic markers (Caspase 3), it was found that all were significantly increased (*p* < 0.05) in the hyperoxia exposure group compared to the RA control group. Nintedanib treatment in the hyperoxia group significantly reduced inflammatory cytokine IL1a levels (Fig. 7a, *p* < 0.01) and TNFa levels (Fig. 7b, *p* < 0.02) as well as the apoptotic marker Caspase 3 levels (Fig. 7c, *p* < 0.01) compared to hyperoxia-exposed rats that did not receive treatment.

## Discussion

BPD is characterized by impaired alveolar and vascular growth and contributes to late mortality and long-term cardiopulmonary morbidities, including persistent respiratory dysfunction and PH,^6,10–13^ yet strategies to prevent BPD and BPD-associated PH are lacking. To determine whether Nintedanib, a tyrosine kinase receptor (TKR) inhibitor, could serve as a novel preventive strategy for BPD, we first examined dose-related effects of Nintedanib on the developing lung in neonatal rats and found that administration of high but not low dose Nintedanib impaired lung airspace growth and caused early death. We then showed that a lower dose Nintedanib preserved lung growth and structure and prevented PH in experimental BPD induced by prolonged hyperoxia. Nintedanib treatment of hyperoxia-exposed rats restored alveolar and vascular growth, improved lung function, and reduced pulmonary artery thickness and RVH. Overall, these findings suggest that Nintedanib preserves lung alveolar and vascular development, improves lung function, and prevents RVH in hyperoxia-exposed rats but demonstrates harmful effects at high doses.

These findings are the first to suggest that Nintedanib treatment may provide a novel strategy for the prevention of BPD and BPD-associated PH after preterm birth. Past preclinical work provided evidence that Nintedanib exhibits striking anti-fibrotic effects, which eventually led to its development for the treatment of patients with idiopathic pulmonary fibrosis (IPF). Data from clinical trials have also shown promising stabilization of lung function.^14–16^ When examining Nintedanib’s utility for BPD treatment, it is imperative to consider the dynamic pathophysiology of BPD. Past histologic studies of infants dying with severe BPD, including patients from the original description of BPD by Northway and colleagues,^1^ have emphasized prominent interstitial fibroproliferative changes in addition to dramatic changes in airway and vascular structure.^28^ In the post-surfactant era, however, improvements in ventilator strategies and respiratory support have prompted recognition of the “new BPD”, which is primarily characterized as lung growth arrest with less fibrosis.^8^ The use of prolonged hyperoxia exposure of infant rats has remained the most commonly used animal model for BPD over the past decades.

Although this model lacks extensive interstitial fibrosis, hyperoxia-induced neonatal lung injury provides consistent inhibition of lung growth that mimics features of clinical BPD, including reduced alveolar growth, abnormal vascular development, and PH, which are key pathologic features of the “new BPD”. As a result, though it is generally considered an anti-fibrotic agent, Nintedanib and its effects in this model are more likely related to its impact as a TKR inhibitor on crucial signaling pathways that are required for normal lung development. At a low dose, Nintedanib promotes alveolar growth and pulmonary function in this model. Preservation of pulmonary vascular growth despite hyperoxia along with the prevention of RVH further suggests that Nintedanib prevents PH and supports the hypothesis that low-dose Nintedanib attenuates acute lung injury and permits sufficient lung repair mechanisms that promote the prevention of BPD.

Large multicenter randomized clinical trials have shown that Nintedanib slows the rate of decline in forced vital capacity (FVC) in adult patients with a diagnosis of IPF with the maximum adult dose set at 300 mg daily.^14–16^ Notably, the dosing in the rat model of lung fibrosis induced by bleomycin administration was starkly different from the dose we used in this study. Nintedanib utilized in this IPF model was administered at a dose of 100 mg/kg daily with treatment starting on day-of-life 7.^29^ Another more recent study that also employed a model of bleomycin-induced pulmonary fibrosis divided the mice treatment groups into low-, medium-, and high-dose Nintedanib which correlated with 30, 60, and 120 mg/kg per day, respectively.^30^ In contrast, administering Nintedanib at a dose of 50 mg/kg in newborn rats proved to be lethal at a strikingly quick rate. This provides insight into the dose-dependent nature of the drug especially in the context of a BPD model versus the IPF model, suggesting that Nintedanib dosing would need to be significantly adjusted in the neonatal population as compared to adults.

While multiple trials have highlighted the efficacy of Nintedanib in adults, studies on anti-fibrotic therapy in pediatric populations have been incredibly limited. One early study, the InPedILD trial, evaluated the efficacy and safety profile of Nintedanib in patients aged 6–17 with childhood interstitial lung diseases using a weight-based dosing regimen.^31^ Results from the trial discovered a reduction in the rate of decline in FVC and stabilization of oxygen saturation at rest in the nintedanib-treated group compared with the placebo group. Regarding adverse effects on pediatric development, no evidence of premature closure of the physes, no clear cases of stunted dentition, and no impact on linear growth were observed throughout this study.^31^ These findings are reassuring; however, the trial was constrained by a small sample size given the rarity of childhood interstitial lung diseases. No studies have been conducted on infants yet, so more work is needed to clarify how Nintedanib precisely acts on lung development in neonates.

Nintedanib traditionally functions as an intracellular TKR inhibitor that competitively binds and inhibits a number of growth factor receptors, including vascular endothelial growth factor receptors (VEGFR), fibroblast growth factor receptors (FGFR), and platelet-derived growth factor receptors (PDGFR).^18^ TKR inhibition subsequently triggers a blockade of downstream signaling cascades, which results in a reduction of fibroblast transformation to myofibroblasts, TGF-B-induced deposition of collagen, neo-angiogenesis, and cell migration and permeability.^18,19,32^ Prior work has shown that Nintedanib extends its effects against non-receptor tyrosine kinase receptors as well, including the Src family kinases.^18,33^ These kinases are integral in signal transduction for a diverse array of cellular processes, including cell migration, differentiation, proliferation, and apoptosis.^33–35^ In vivo studies have indicated that Src family kinase inhibition also attenuates collagen accumulation and other markers of lung fibrosis.^33,34^

Based on this mechanistic framework, we postulate that high-dose Nintedanib could be exerting inhibitory actions against a broader range of growth factor receptors that are required for lung development. With higher doses, it appears that Nintedanib more widely disrupts signaling transduction pathways that are foundational for normal pulmonary structure and function, which results in significant lethality. In contrast, we think that low-dose Nintedanib exerts therapeutic effects due to more specific inhibition of growth factor receptors that are essential for the proliferation and survival of myofibroblasts.^18^ It is potentially mitigating the aberrant lung repair response through selective inhibition of receptors, resulting in the preservation of normal alveolarization and prevention of pathologic lung vasculature and structural remodeling. This could explain its role in promoting profound pulmonary recovery in hyperoxia-exposed rats with no adverse effects.

Since Nintedanib has been known to target the Src family of non-tyrosine kinases, we analyzed protein expression of Src in day 5 rat lungs to determine whether this could be a possible receptor pathway that Nintedanib was downregulating to stimulate pulmonary recuperation. Western blot data revealed no significant changes in Src kinase phosphorylation between the hyperoxia-exposed rats and Nintedanib-treated hyperoxia-exposed rats. However, while Nintedanib does not appear to alter Src kinase activity in this model, this finding does not exclude the possibility that Nintedanib could still impact alternative tyrosine or non-tyrosine growth factor receptors to support proper lung development. Additionally, outside of these traditional fibrotic mechanisms, Nintedanib also attenuates inflammatory cytokines and apoptotic markers that are damaging to the developing lung. Thus, Nintedanib could both mitigate the initial hyperoxic damage to the lung tissue and subsequently prevent the aberrant lung repair response from occurring.

Potential limitations of the study include challenges in identifying a rodent model that fully encapsulates the long-term fibrosis associated with BPD, as touched on earlier. Exposure to high oxygen concentrations is one of the most critical elements contributing to the progression of BPD.^14,17^ The duration of hyperoxia exposure in the animal model used in this study produced lung morphological pathology that mimics multiple features of BPD and associated pulmonary hypertension, but again, this is not a BPD model that effectively reproduces fibrosis. Still, the increased respiratory resistance and decreased compliance seen in the hyperoxia model may indicate some element of altered extracellular matrix at play, and the data collected suggests that Nintedanib could be acting as more than simply an anti-fibrotic agent. Further work is needed to define the mechanisms through which Nintedanib preserves lung growth in the experimental BPD model. We also speculate that there are extra-pulmonary and extra-cardiac effects that were not addressed in this study, though future studies may consider exploring the effects of Nintedanib on other organ systems as well.

Thus, we conclude that high doses of Nintedanib impairs lung growth and alveolarization and increase mortality in neonatal rats; however, lower doses of Nintedanib improve lung growth and function, increase pulmonary vessel density, and prevent PH in hyperoxia-exposed rat pups. We speculate that Nintedanib may provide a novel strategy for the prevention and treatment of BPD, but in light of the adverse effects on survival and lung growth at higher doses, there is a need for caution with future clinical studies in infants and younger children with developmental lung disorders, including BPD.

## Data Availability

All data generated or analysed during this study are included in this published article.
